# The Impact of a Healthy Lifestyle on Psychological Well-Being Among Saudi Adolescent Girls Attending Secondary Schools in Taif City, Saudi Arabia

**DOI:** 10.7759/cureus.50189

**Published:** 2023-12-08

**Authors:** Sarah S Bin Baz, Waad M Malibarey, Hazim A Alsalmi, Mohammed D Alzaydi, Alwah M Alqahtani, Reham Y Alghamdi

**Affiliations:** 1 Family Medicine, Ministry of Health, Riyadh, SAU; 2 Family Medicine, Ministry of Health, Taif, SAU; 3 Family Medicine, Family Medicine Program, Ministry of Health, Taif, SAU; 4 Family Medicine, Ministry of Health, Jeddah, SAU

**Keywords:** stress, anxiety, depression, schoolgirls, lifestyle

## Abstract

Background and objective

A healthy lifestyle encompasses healthy eating, regular exercise, getting enough sleep, and avoiding smoking, drug abuse, and alcohol, which will help improve mental health and manage the symptoms of anxiety, stress, and depression. In this study, we aimed to evaluate the relationship between a healthy lifestyle and psychological well-being among Saudi adolescent girls.

Materials and methods

A cross-sectional study was conducted among adolescent girls in secondary schools in Taif City, Saudi Arabia. The main outcome measures were the Simple Lifestyle Indicator Questionnaire (SLIQ), Patient Health Questionnaire-2 (PHQ-2) and PHQ-9 (if PHQ-2-positive), Generalized Anxiety Disorder 7-item (GAD-7) questionnaire, and Perceived Stress Scale (PSS) scores.

Results

The age of the respondents ranged between 15 and 19 years with an arithmetic mean of 16.72 and a standard deviation (SD) of 0.96 years. Most of the students (58.3%) followed a healthy lifestyle whereas only 6.7% followed an unhealthy one. The prevalence of depression was 52.5%; moderately severe depression was observed in 14.8% and severe depression was seen in 6.9% of the schoolgirls. Moderate or severe anxiety was observed in 24.3% and 17.8% of schoolgirls, respectively. High perceived stress was observed in 18% of the students. There was a statistically significant association between students' lifestyle and the severity of depression, anxiety, and perceived stress (p<0.001).

Conclusion

While unhealthy lifestyles are not common among secondary schoolgirls in Taif City, we found a significant association between such lifestyles among students and the deterioration of their psychological well-being.

## Introduction

Health is important to all of us. According to the World Health Organization (WHO), health is a state of complete physical, mental, and social well-being and not just the absence of disease or infirmity. There are different types of health: mental, physical, spiritual, emotional, and financial. All of these contribute to overall health, decrease stress, and improve mental and physical well-being [1]. Healthy nutrition does not mean tasteless food; on the contrary, it is a method to ensure eating all essential nutrients to maintain the body's function properly. The definition of a balanced diet varies from person to person and depends on many factors like age, gender, lifestyle, and medical conditions. Following a balanced and healthy eating lifestyle keeps the body in excellent and optimum condition [2]. When it comes to nutrition, there are macronutrients and micronutrients. The "big 3" macronutrients (macros) are carbohydrates, protein, and fats; fiber can also be considered a macronutrient. These macronutrients can help improve weight, health, and overall physical well-being [2].

Carbohydrate is an essential source of energy. It is classified into simple (sugar) and complex starches. Carbohydrates from grain are classified into whole and refined grain; it is healthier to eat whole grain because it has a milder effect on blood sugar and insulin than refined grains. The recommended dietary intake of carbohydrates is 45-65% of the total calorie intake. Added sugars should constitute less than 10% of total calories [2]. Protein is an essential part of a healthy diet. It helps build muscles, cartilage, and skin and also plays a role in hormonal regulation. The recommended dietary intake of proteins is 10-35% of the total calorie intake [2]. Fat is also an essential part of a healthy balanced diet. It helps the body to absorb fat-soluble vitamins like vitamin A, vitamin D, vitamin E, and vitamin K. Also, fat is a rich source of energy. There are three types of fat: saturated, unsaturated, and trans-fat; as part of maintaining a healthy diet, it is advisable to replace saturated and trans-fat with unsaturated fat. The recommended dietary intake of fats is 20-35% of the total calorie intake [2]. Fibers are found mainly in fruits, vegetables, whole grains, and legumes. Fibers provide a lot of benefits; they help prevent and relieve constipation, help maintain a healthy weight, and lower the risk of diabetes, heart disease, and some types of cancer. There are two types of fibers: soluble and insoluble. Soluble fiber helps lower cholesterol and glucose levels. Insoluble fibers help to relieve and prevent constipation. The recommended dietary intake of fiber is 14 g per 1000 calories. For most moderately active adults in the United States, that translates to approximately 25-36 g per day [2]. Micronutrients include vitamins and minerals. These nutrients are indispensable for numerous vital functions in the human body and it is necessary to consume them through dietary sources [3].

Physical activity is defined as any body movement produced by skeletal muscles that requires energy expenditure. Regular physical activities confer a lot of health benefits. They help to prevent and manage non-communicable diseases like cardiovascular disease, hypertension, diabetes, and several types of cancers. They help to maintain a healthy body weight and improve mental health and quality of life. The recommended physical activity for an adult involves 150-300 minutes of moderate-intensity aerobic exercise or at least 75-150 minutes of vigorous-intensity aerobic exercise, or an equivalent combination of moderate- and vigorous-intensity exercise regimens throughout the week. They should also perform muscle-strengthening activities that involve all major muscle groups two or more days a week. For children and adolescents aged 5-17 years, the recommended physical activity involves at least 60 minutes per day of moderate-to-vigorous intensity, mostly aerobic, exercise across the week [4].

Smoking can significantly harm the body. A smoker is at a significant risk of developing diseases that affect several organs in the body. It increases the risk of cardiovascular diseases like acute coronary syndrome (ACS) and stroke. It can also cause lung diseases like COPD, increase the risk of developing different types of cancers like laryngeal cancer, lung cancer, and bladder cancer, and increase the risk of infertility in both men and women. It also affects the immune system. Quitting smoking is necessary to improve health and well-being [5]. Psychological well-being is defined as a state that combines feeling good and functioning effectively. It does not mean feeling good all the time; the experience of negative emotions is also a normal part of life, and managing these negative or painful emotions is vital for long-term well-being. Psychological well-being is affected when negative emotions are extreme or long-lasting and interfere with daily activities [6].

WHO defines adolescents as individuals aged between 10 and 19 years. Adolescence is a period of life with specific health and developmental needs and rights. Also, it is a time to develop knowledge and skills, learn to manage emotions and relationships, and acquire attributes and abilities that will be important for enjoying the adolescent years and laying the foundation for assuming the role of an adult [7]. This study aimed to evaluate the association between a healthy lifestyle and psychological well-being among adolescent girls in Taif City, Saudi Arabia.

## Materials and methods

A cross-sectional study was conducted in Taif City, which is located in the Makkah Province of Saudi Arabia. The city has an estimated population of 688,693. There are 72 female-only secondary schools in Taif City (22 schools in the Al-Hawiyah region, 29 schools in the West region, and 21 schools in the East region). This study included all adolescent girls attending secondary schools in Taif City (n=18,609).

By using the Raosoft equation with a confidence interval of 95%, a margin of error of 5%, and a distribution response of 50%, the determined sample size was 377. A multistage random sampling technique was adopted. In the first stage, female secondary schools in Taif City were divided into three groups according to their geographic location (East, West, and Al-Hawiyah). In the second stage, two to three schools were selected randomly from each region. In the third stage, an almost equal number of students were selected from nine schools; therefore, about 70-75 students were selected from each school. In the fourth stage, 25 students were chosen randomly from every level. Students who were already diagnosed with psychiatric illnesses (anxiety, depression, or others) were excluded from the study.

Four self-administered, valid, and reliable questionnaires were distributed and collected by the researcher: the Simple Lifestyle Indicator Questionnaire (SLIQ), Patient Health Questionnaire-2 (PHQ-2) and PHQ-9 (if PHQ-2-positive), Generalized Anxiety Disorder 7-item (GAD-7) questionnaire, and Perceived Stress Scale (PSS) [11]. The validity of the Arabic version of SLIQ was ascertained by two family medicine consultants.

The questionnaire included sociodemographic data: the name of the school, age, scholastic level, GPA (general grade point average), height, weight, and body mass index (BMI) calculated by the researcher, and medical history of psychiatric illness diagnosed by a doctor (depression, anxiety, or other). BMI was calculated as weight in kilograms divided by the square of height in meters. As per the CDC guidelines, the BMI-for-Age Percentile Growth Chart was used [12]. SLIQ consists of 12 questions on five domains (diets, physical activity, smoking, stress, and alcohol consumption). The overall SLIQ score is determined by summing up the raw scores of the five categories. The students were classified as follows based on the total scores: unhealthy (0-4), intermediate (5-7), and healthy (8-10) [8]. PHQ-2 contains the two first questions of PHQ-9, and if it was found positive, PHQ-9 was used. The level of depression in students was categorized as follows as per the total scores: minimal depression (1-4), mild depression (5-9), moderate depression (10-14), moderately severe (15-19), and severe (20-27) [13]. The GAD-7 comprises seven questions. The anxiety level was categorized as follows based on the total scores: mild anxiety (5-9), moderate anxiety (10-14), and severe anxiety (15+) [14]. PSS consists of 12 questions. There are two subscales in the PSS-10: (1) perceived helplessness (items 1, 2, 3, 6, 9, and 10) - measuring an individual’s feelings of a lack of control over their circumstances or their own emotions or reactions; and (2) lack of self-efficacy (items 4, 5, 7, and 8) - measuring an individual’s perceived inability to handle problems. To calculate the total PSS score, responses to the four positively stated items (items 4, 5, 7, and 8) first need to be reversed, and the stress level is categorized as follows based on the total score: low stress (0-13), moderate stress (14, 26), and high perceived stress (27, 40) [11].

Written permission from the authority and ethical approval from the concerned body (the Research and Studies Department of the Directorate of Health Affairs in Taif) was obtained before the commencement of the study. The data were collected and verified manually and then coded before being entered into the computer. SPSS Statistics software version 28.0 was used for data entry and analysis. Frequency and percentage were applied to describe categorical variables whereas range, arithmetic mean, and standard deviation (SD) were used to describe continuous numerical variables. The chi-square test was utilized to test for the association between categorical variables, and a p-value of less than 0.05 was considered statistically significant.

## Results

A total of 562 schoolgirls were initially recruited. However, 23 were excluded either due to incomplete questionnaires or because of being previously diagnosed with psychological diseases (depression and anxiety). Thus, a total of 539 students were included in the final analysis. The age of the participants ranged between 15 and 19 years with an arithmetic mean of 16.72 and a standard deviation of 0.96 years. They were almost equally distributed between the three educational levels. While a majority of them (83.3%) had an excellent GPA, 13.9% had a very good GPA (Table 1).

**Table 1:** Personal characteristics of the participants (n=539) SD: standard deviation; GPA: grade point average

Variables	Values
Age, years	
Range	15-19
Mean ±SD	16.72 ±0.96
Scholastic level, n (%)	
First secondary	175 (32.5)
Second secondary	183 (34)
Third secondary	181 (33.6)
GPA, n (%)	
Weak	0 (0)
Pass	2 (0.4)
Good	13 (2.4)
Very good	75 (13.9)
Excellent	449 (83.3)

**Figure 1:**
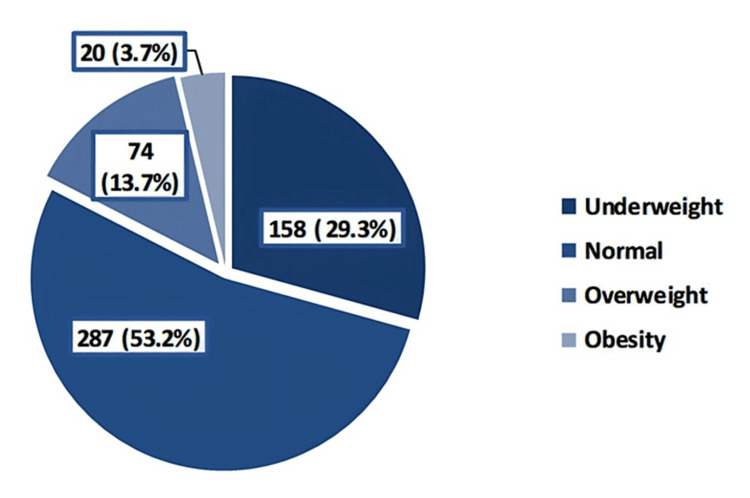
Distribution of the subjects by body mass index

**Figure 2:**
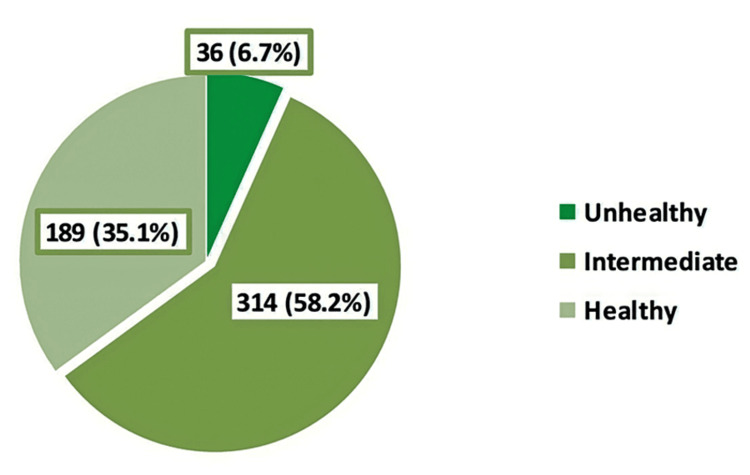
Classification of participants' lifestyle based on the Simple Lifestyle Indicator Questionnaire score

Overweight and obesity were observed among 13.7% and 3.7% of the schoolgirls, respectively (Figure 1). While most of the students followed a healthy lifestyle (58.2%), only 6.7% followed an unhealthy lifestyle (Figure 2).

**Figure 3:**
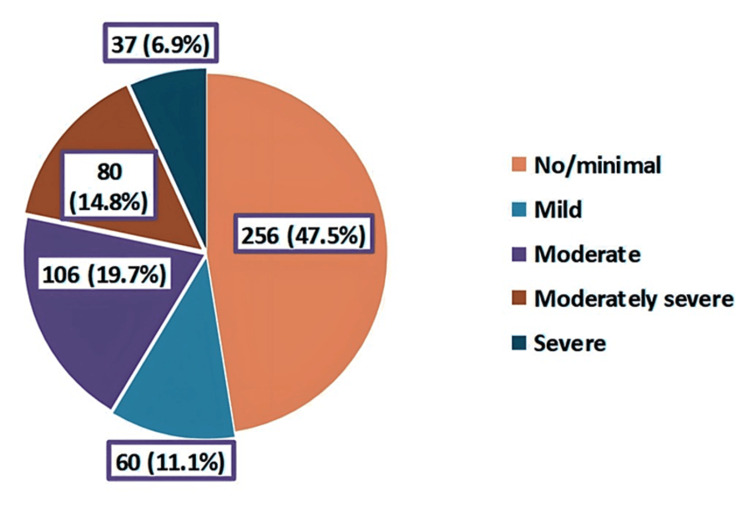
Classification of the level of depression among the participants based on Patient Health Questionnaire (PHQ)-2 and PHQ-9

**Figure 4:**
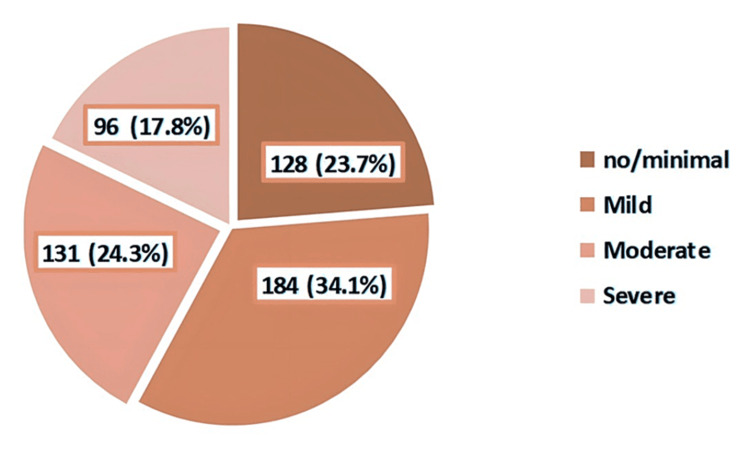
Classification of anxiety levels among the participants based on Generalized Anxiety Disorder (GAD)-7 questionnaire

**Figure 5:**
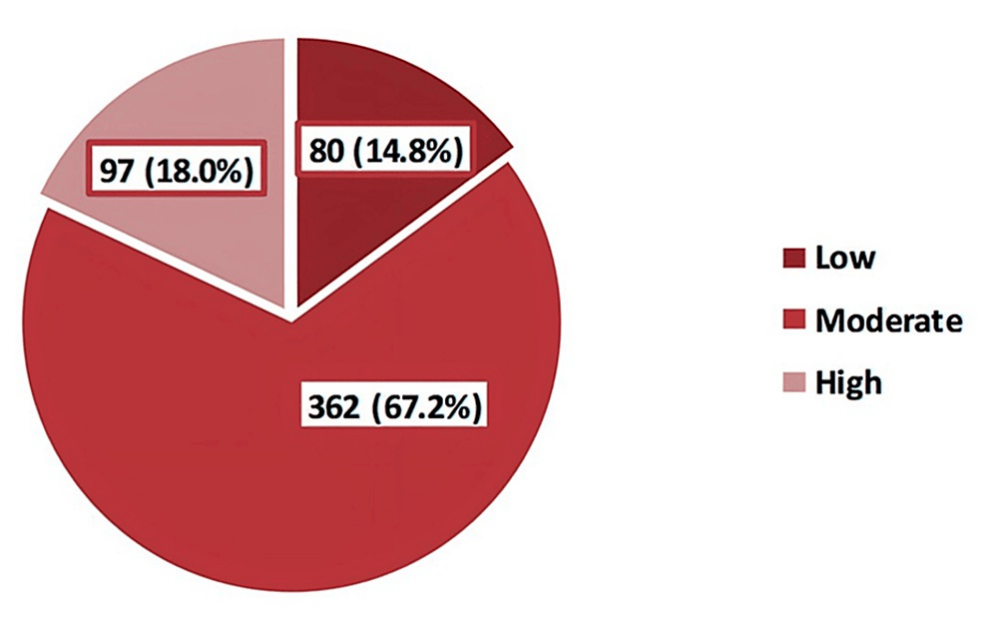
Classification of perceived stress among the participants according to the Perceived Stress Scale (PSS)

The prevalence of depression among schoolgirls was 52.5%; while 14.8% had a moderately severe level of depression, 6.9% were severely depressed (Figure 3). As for anxiety levels, we found that while 24.3 of schoolgirls had moderate anxiety, 17.8% had severe anxiety levels (Figure 4). Regarding stress, high perceived stress was observed among 18% of the participants (Figure 5).

The mean age of students with unhealthy lifestyles (16.89 ±0.98 years) was significantly higher than those with intermediate (16.84 ±0.91 years) and healthy lifestyles (16.50 ±0.98 years) (p<0.001). Also, 47.4% of students in the first scholastic level were found to have a healthy lifestyle compared to 26.5% in the third scholastic level (p<0.001) (Table 2).

**Table 2:** Association between personal characteristics of the students and their lifestyle SD: standard deviation

Variables	Lifestyle			P-value
	Unhealthy (n=36)	Intermediate (n=314)	Healthy (n=189)	
Age, years, mean ±SD	16.89 ±0.98	16.84 ±0.91	16.50 ±0.98	<0.001
Scholastic level, n (%)				
First (n=175)	7 (4.0)	85 (48.6)	83 (47.4)	<0.001
Second (n=183)	12 (6.6)	113 (61.7)	58 (31.7)	
Third (n=181)	17 (9.4)	116 (64.1)	48 (26.5)	

Association between students' lifestyle and their psychological well-being

Depression

Students with unhealthy lifestyles were more likely to have moderately severe depression (27.8%) or severe depression (8.3%) compared to those with healthy lifestyles, in whom the rates of moderately severe and severe depression were 9% and 2.6%, respectively (p<0.001) (Table 3).

**Table 3:** Association between students' lifestyle and severity of depression Pearson chi-square value: 36.61; degree of freedom: 8; p<0.001

Lifestyle	Depression, n (%)				
	No/minimal (n=256)	Mild (n=60)	Moderate (n=106)	Moderately severe (n=80)	Severe (n=37)
Unhealthy (n=36)	9 (25.0)	3 (8.3)	11 (30.6)	10 (27.8)	3 (8.3)
Intermediate (n=314)	130 (41.5)	35 (11.1)	67 (21.3)	53 (16.9)	29 (9.2)
Healthy (n=189)	117 (62.0)	22 (11.6)	28 (14.8)	17 (9.0)	5 (2.6)

Anxiety

Moderate and severe anxiety were reported in 38.8% and 27.8% of students with unhealthy lifestyles compared to 16.4% and 12.2% respectively of students with healthy lifestyles (p<0.001) (Table 4).

**Table 4:** Association between students' lifestyle and severity of anxiety Pearson chi-square value: 37.58; degree of freedom: 6; p<0.001

Lifestyle	Anxiety, n (%)			
	No/minimal (n=128)	Mild (n=184)	Moderate (n=131)	Severe (n=96)
Unhealthy (n=36)	1 (2.8)	11 (30.6)	14 (38.8)	10 (27.8)
Intermediate (n=314)	59 (18.8)	106 (33.7)	86 (27.4)	63 (20.1)
Healthy (n=189)	68 (36.0)	67 (35.4)	31 (16.4)	23 (12.2)

Perceived Stress

More than one-third (38.9%) of students with unhealthy lifestyles had high levels of perceived stress compared to only 9.5% of those with healthy lifestyles (p<0.001) (Table 5).

**Table 5:** Association between students' lifestyle and severity of perceived stress Pearson chi-square value: 43.56; degree of freedom: 4; p<0.001

Lifestyle	Perceived stress, n (%)		
	Low (n=80)	Moderate (n=362)	High (n=97)
Unhealthy (n=36)	1 (12.8)	21 (58.3)	14 (38.9)
Intermediate (n=314)	30 (9.6)	219 (69.7)	65 (20.7)
Healthy (n=189)	49 (25.9)	122 (64.6)	18 (9.5)

Association between students' body mass index and their psychological well-being

Depression

There was no statistically significant association between students' body mass index and their severity of depression (Table 6).

**Table 6:** Association between students' body mass index and severity of depression Pearson chi-square value: 9.62; degree of freedom: 12; p=0.649

Body mass index category	Depression, n (%)				
	No/minimal (n=256)	Mild (n=60)	Moderate (n=106)	Moderately severe (n=80)	Severe (n=37)
Underweight (n=158)	79 (50.0)	16 (10.1)	30 (19.0)	24 (15.2)	9 (5.7)
Normal (n=287)	139 (48.4)	35 (12.2)	56 (19.5)	39 (13.6)	18 (6.3)
Overweight (n=74)	32 (43.2)	7 (9.5)	15 (20.3)	14 (18.9)	6 (8.1)
Obesity (n=20)	6 (30.0)	2 (10.0)	5 (25.0)	3 (15.0)	4 (20.0)

Anxiety

There was no statistically significant association between students' body mass index and their severity of anxiety (Table 7).

**Table 7:** Association between students' body mass index and severity of anxiety Pearson chi-square value: 14.26; degree of freedom: 9; p=0.113

Body mass index category	Anxiety, (%)			
	No/minimal (n=128)	Mild (n=184)	Moderate (n=131)	Severe (n=96)
Underweight (n=158)	38 (24.1)	57 (36.0)	37 (23.4)	26 (16.5)
Normal (n=287)	74 (25.8)	101 (35.2)	67 (23.3)	45 (15.7)
Overweight (n=74)	14 (18.9)	23 (31.1)	18 (24.3)	19 (25.7)
Obesity (n=20)	2 (10.0)	3 (15.0)	9 (45.0)	6 (30.0)

Perceived Stress

There was no statistically significant association between students' body mass index and the severity of their perceived stress (Table 8).

**Table 8:** Association between students' body mass index and severity of perceived stress Pearson chi-square value: 10.06; degree of freedom: 6; p=0.122

Body mass index category	Perceived stress, n (%)		
	Low (n=80)	Moderate (n=362)	High (n=97)
Underweight (n=158)	23 (14.6)	105 (66.4)	30 (19.0)
Normal (n=287)	36 (12.5)	205 (71.5)	46 (16.0)
Overweight (n=74)	17 (23.0)	42 (56.7)	15 (20.3)
Obesity (n=20)	4 (20.0)	10 (50.0)	6 (30.0)

Association between lifestyle and scholastic achievement

There was no statistically significant association between students' lifestyle and their scholastic achievement expressed as GPA (Table 9).

**Table 9:** Association between students' lifestyle and their grade point average Pearson chi-square value: 9.82; degree of freedom: 6; p=0.133

Lifestyle	Grade point average, n (%)			
	Pass (n=2)	Good (n=13)	Very good (n=75)	Excellent (n=449)
Unhealthy (n=36)	1 (2.8)	1 (2.8)	7 (19.4)	27 (75.0)
Intermediate (n=314)	1 (0.3)	10 (3.2)	42 (13.4)	261 (83.1)
Healthy (n=189)	0 (0.0)	2 (1.1)	26 (13.8)	161 (85.1)

## Discussion

The psychological well-being and mental health of adolescents are influenced by numerous factors, including their lifestyles, which they can change. However, many adolescents ignore the utmost importance of different components of a healthy lifestyle such as exercise, healthy eating, and avoiding smoking, which could help them improve their psychological well-being [15]. The present study aimed to evaluate the association between a healthy lifestyle and psychological well-being among schoolgirls attending secondary schools in Taif City, Makkah Province, Saudi Arabia.

Our findings revealed that a considerable proportion of schoolgirls had severe forms of psychological problems: 14.8% and 6.9% of them had moderately severe and severe depression, respectively; 17.8% had severe anxiety; and 18% had high perceived stress. This indicates that these students do have psychological issues. In a similar study performed in Malaysia among university students [16], no students were categorized as having severe forms of depression, anxiety, and stress while >20% of them had moderate levels. However, the comparison between the two studies should factor in the difference in the demographics of the participants, as the present study included secondary school girls. In contrast, the Malaysian study included university students of both genders. Also, the perception of psychological elements could differ between the two countries.

The current study revealed that a minority of the students (6.7%) followed unhealthy lifestyles while a greater proportion (58.3%) followed a healthy lifestyle. In a similar study conducted in Malaysia using the same data collection tool utilized in the present study, the majority of the students (71.6%) were classified as having an unhealthy lifestyle with 28.3% having an intermediate healthy lifestyle [16]. The great differences between the two studies should be seen in light of cultural and environmental differences as well as variations between the sociodemographic characteristics of the participants.

Furthermore, in this study, we observed an association between the unhealthy lifestyle of schoolgirls and psychological issues among them in the form of depression, anxiety, and stress. This indicates that maintaining a healthy lifestyle is essential for adolescents in terms of their mental health. Other recent studies have reported similar findings. Among adolescents in Belgium, healthy lifestyle behaviors were associated with better psychological and mental health [17]. In Malaysia, students with poor lifestyles had higher anxiety, depression, and stress [16]. In Nigeria, moderate to high levels of physical activity (which is an element of a healthy lifestyle) were associated with a high level of psychological well-being among male university students [18 ]. The same has been observed in Finland where higher physical fitness and leisure-time physical activity levels were observed to promote specific dimensions of health-related quality of life while morbidities impaired them among the young population [19]. A study from Iran has suggested that modifying the lifestyle of university students to adopt healthier ways can improve their mental health [20]. In Namibia, it has been observed that school students engaging in a more significant amount of harmful, consumptive, sedentary, and risky sexual behaviors was associated with an elevated risk for anxiety, suicide attempts, and loneliness [21]. A study conducted in Australia reported an association between lifestyle behaviors and mental health among schoolchildren [22]. In New Zealand and Australia, mental health in adolescents was found to improve with practicing aerobic exercise at lunchtime three times per week for eight weeks [23]. A study carried out in the UK reported that physical activity was important in improving mental well-being in students [24]. Even at the local level, as evidenced in a study in Abha, Saudi Arabia, the findings supported an association between adolescents' lifestyle-related behaviors and their quality of life [25].

In this study, older students and those of higher scholastic level were more likely to have an unhealthy lifestyle. This is an alarming finding, and it indicates that scarce efforts are made in schools as well as homes to encourage students to follow a healthy lifestyle, and the consequences of such lack of effort tend to aggravate with time. However, another study carried out in Malaysia [16] found no significant association between a healthy lifestyle and the year of study. In the present study, we did not observe a significant association between students' lifestyle and their scholastic performance. However, in another study carried out in Malaysia, students with an unhealthy lifestyle were more likely to have difficulty in learning and thus had poorer scholastic achievement and lower school attendance [26].

Some studies have reported an association between body mass index of adolescents and their psychological well-being [27]. A study from the Netherlands observed that overweight and obesity were significantly associated with mental health problems in adolescents, and victimization played a mediating role in this association [28]. In New Zealand, lower health-related quality of life and a higher prevalence of psychological difficulties were more prevalent in obese children and adolescents [29]. In the United States, a study found that schoolchildren who believed that it was difficult to live a healthy lifestyle were more likely to be overweight [30]. However, the present study failed to find such an association.

This study has several limitations, primarily its cross-sectional design, which precluded us from ascertaining the causal directions of the associations. Also, the study was conducted only among schoolgirls, and hence its findings cannot be compared with those in studies among males. Moreover, we did not analyze each aspect of lifestyle as a separate domain, as we used the total score of lifestyle categories for comparisons. Despite these shortcomings, the fact that we included a large sample size and applied multi-stage random sampling can be considered the major strengths of this study, which enables the generalizability of our findings.

## Conclusions

Based on our findings, while unhealthy lifestyles were not common among secondary schoolgirls in Taif, those were associated with the deterioration of their psychological well-being. Unhealthy lifestyles were more prevalent among older students and those of higher scholastic levels. Overweight and obesity affected a relatively small proportion of schoolgirls and these were not associated with their mental health. Also, students' lifestyles were not associated with their scholastic performance. We recommend that schools should offer and promote healthy activities to encourage healthy lifestyle practices among students. In addition, regular screening for the psychological well-being of school students using a simple valid tool should be implemented. Also, the inclusion of mental health promotion programs in school curricula should be considered besides promoting healthy lifestyles. Finally, further studies that include male students and employ a longitudinal design are warranted to gain deeper and broader insights into the impact of healthy lifestyle behaviors on the mental health of students.
